# Potential of Matrix Metalloproteinase Inhibitors for the Treatment of Local Tissue Damage Induced by a Type P-I Snake Venom Metalloproteinase

**DOI:** 10.3390/toxins12010008

**Published:** 2019-12-20

**Authors:** Lina María Preciado, Jaime Andrés Pereañez, Jeffrey Comer

**Affiliations:** 1 Programa de Ofidismo/Escorpionismo, Facultad de Ciencias Farmacéuticas y Alimentarias, Universidad de Antioquia UdeA, Calle 70 No. 52-21, Medellín 050010, Colombia; linampr@gmail.com; 2 Institute of Computational Comparative Medicine, Kansas State University, Manhattan, KS 66506, USA

**Keywords:** local tissue damage, molecular dynamics, inhibitors, peptidomimetics, snake venom metalloproteinase, free energy calculation

## Abstract

Snake bite envenoming is a public health problem that was recently included in the list of neglected tropical diseases of the World Health Organization. In the search of new therapies for the treatment of local tissue damage induced by snake venom metalloproteinases (SVMPs), we tested the inhibitory activity of peptidomimetic compounds designed as inhibitors of matrix metalloproteinases on the activities of the SVMP Batx-I, from *Bothrops atrox* venom. The evaluated compounds show great potential for the inhibition of Batx-I proteolytic, hemorrhagic and edema-forming activities, especially the compound CP471474, a peptidomimetic including a hydroxamate zinc binding group. Molecular dynamics simulations suggest that binding of this compound to the enzyme is mediated by the electrostatic interaction between the hydroxamate group and the zinc cofactor, as well as contacts, mainly hydrophobic, between the side chain of the compound and amino acids located in the substrate binding subsites S1 and S1${}^{\prime }$. These results show that CP471474 constitutes a promising compound for the development of co-adjuvants to neutralize local tissue damage induced by snake venom metalloproteinases.

## 1. Introduction

Snake bite envenoming is a major public health problem, which the World Health Organization has recognized as a Category A neglected tropical disease since 2017 [1]. Envenoming causes mortality and morbidity mainly in tropical and subtropical areas, such as sub-Saharan Africa, South and Southeast Asia, Papua New Guinea and Latin America [2], with annual deaths estimated at 100,000 and sequelae in more than 400,000 cases [3]. In Latin America, recent estimates suggest about 57,500 annual envenomings, with an incidence of 6.2 per 100,000 people [2]. In Colombia specifically, 5434 cases were reported in 2018, with an incidence of 10.9 per 100,000 people [4]. It is estimated that snakes of the *Bothrops* genus (Viperidae), *B. asper* and *B. atrox*, cause about 70–90% of all envenomings in Colombia.

Envenoming by *Bothrops* species is characterized by prominent local tissue damage, which causes pain, edema, blistering, hemorrhage, and myonecrosis [5]. In moderate and severe cases, there are also systemic manifestations characterized mainly by hemorrhage, coagulopathies, acute renal failure and cardiovascular shock [6]. These effects are induced by the main toxins of the venom: snake venom metalloproteinases (SVMPs), serine proteinases and phospholipases A${}_{2}$ [7].

**Figure 1:**
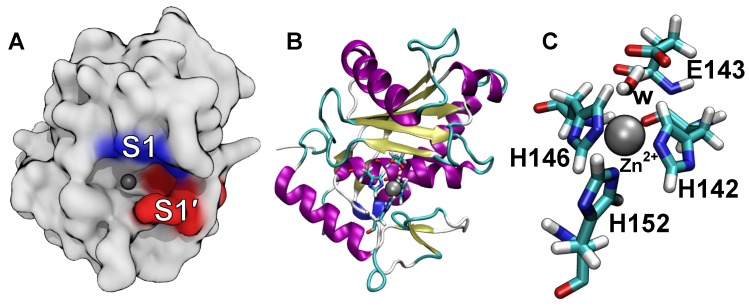
Structure of the metalloproteinase BaP1. (**A**) Location of the catalytic zinc cofactor and S1 and S1${}^{\prime }$ subsites in BaP1. The zinc cofactor is shown as a gray sphere. (**B**) Secondary structure representation for the same orientation of BaP1 shown in panel A. Residues in the active site are explicitly represented. (**C**) Detailed structure of the metalloproteinase active site. The zinc cofactor is tetrahedrally coordinated by the Nϵ2 atoms of three histidine residues (H142, H146, H152) and the oxygen atom of the catalytic water molecule (w). This water molecule is coordinated by the highly conserved residue E143. H, C, N, and O atoms are shown in white, aquamarine, blue, and red, respectively. The zinc cofactor is represented as a gray sphere. (PDB code 2W15).

The SVMPs are zinc-dependent endopeptidases, with a molecular masses between 20 and 100 kDa. These enzymes are members of the subfamily of proteinases known as metzincins. They contain a consensus zinc binding sequence, HEXXHXXGXX, and a 1–4 turn containing a highly conserved methionine residue that underlies the three histidine residues and zinc cofactor involved in the catalysis [8]. SVMPs are classified as type P-I, P-II, or P-III, depending on domain organization. P-I SVMPs consist only of a proteinase domain, while P-II SVMPs possess proteinase and disintegrin domains. P-III SVMPs have proteinase, disintegrin, and cysteine rich domains [9]. BaP1, a P-I metalloproteinase isolated from the venom of *B. asper* inhabiting the Pacific region of Costa Rica, has been extensively studied since its crystallographic structure was reported [10]. P-I type metalloproteinases constitute about 30% of Pacific *B. asper* venom proteins [11]. The structure of the BaP1 is shown in Figure 1. When a polypeptide is hydrolyzed by the SVMP, its scissile peptide bond is positioned near the zinc cofactor of the metalloproteinase and the residues preceding and following this bond make contact, respectively, with the S1 and S1${}^{\prime }$ subsites (Figure 1A) [12,13]. The residues of BaP1 in the active site, as well as the catalytic water molecule, are highlighted in Figure 1B,C.

The primary biological activity of SVMPs is local and systemic hemorrhage, mediated by proteolytic degradation of extracelullar matrix proteins of capillary vessels, such as type IV collagen, nidogen, laminin and perlecan [14]. Additionally, these enzymes induce myotoxicity, inflammation, edema and blistering [15], effects that are difficult to neutralize by antivenoms due to their rapid onset, especially if there is a delay in serotherapy administration [16]. In May 2019, the World Health Organization launched a plan of work with the goal of reducing incidence of snakebite death and disability by half, through strengthening of health systems and the provision of safe and effective treatments [17]. This plan directly promotes research and development for small-molecule SVMP inhibitors that could be injected in the field.

To the date, the most promising inhibitor of SVMPs is a synthetic hydroxamate peptidomimetic known as batimastat. This compound was initially developed as a matrix metalloproteinase (MMP) inhibitor to treat cancer [18] and later demonstrated neutralization of the biological activities of the snake venom metalloproteinase BaP1, from *B. asper* venom. Specifically, batimastat inhibited the proteolytic, hemorrhagic, dermonecrotic, and edema forming activities of BaP1 when it was injected intramuscularly at the site of BaP1 injection immediately following injection of the latter. However, this inhibition was reduced when there was a time lag between the injection of BaP1 and the injection of batimastat [19]. Similarly, it was determined that batimastat inhibits *B. asper* venom lethality when a venom challenge dose of double the LD${}_{50}$ was administered by intraperitoneal and intravenous routes, with ED${}_{50}$ values of 250 and 22 μM, respectively. In addition, this compound exerted a significant inhibition of in vitro coagulant and in vivo defibrinogenating activities of the venom, demonstrating its ability to neutralize the systemic effects induced by *B. asper* venom [20].

Recently, the peptidomimetic hydroxamate molecules batimastat and marimastat were shown to inhibit local and pulmonary hemorrhagic, coagulant, defibrinogenating, and proteinase activities of *Echis ocellatus* venom in pre-incubation assays [21], demonstrating the potential of synthetic matrix metalloproteinase inhibitors as therapeutic tools against snake bite envenoming.

Therefore, in this study we evaluated the potential of four commercial MMP inhibitors, 4-aminobenzoyl-Gly-Pro-D-Leu-D-Ala hydroxamic acid (4AM), ARP-100 (ARP), CP471474 (CP) and phosphoramidon (PH) [22,23,24,25] selected from previous molecular docking studies, for the inhibition of proteolytic, hemorrhagic and edema forming activities of the metalloproteinase Batx-I isolated from *B. atrox* venom from Colombia. The mechanisms of inhibition were studied by the computational techniques molecular docking and molecular dynamics.

**Figure 2:**
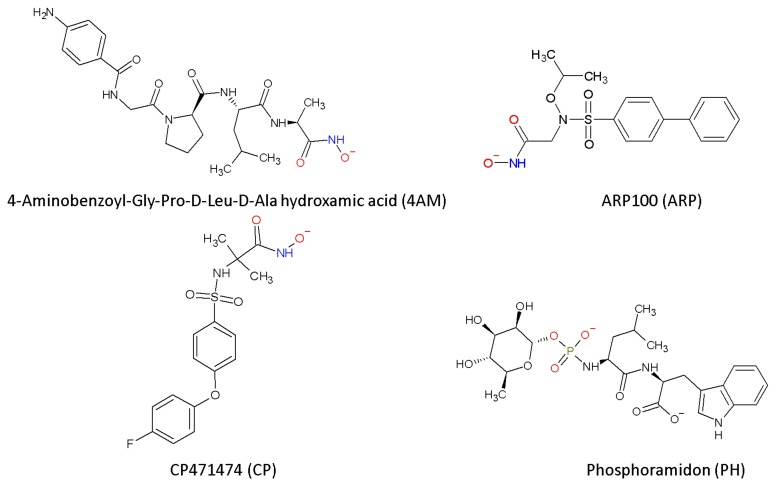
Chemical structures of the peptidomimetic compounds studied in this work at physiological pH.

ARP-100 (ARP) is a hydroxamate peptidomimetic with a sulfonamide group and a biphenyl moiety that has been reported as a MMP-2-preferring inhibitor with an IC${}_{50}$ value of 12 nM [23]. 4-aminobenzoyl-Gly-Pro-D-Leu-D-Ala hydroxamic acid (4AM) is a tetrapeptidyl hydroxamic acid; it is alternatively called MMP Inhibitor I (IC${}_{50}$ = 1.0 μM). It also inhibits MMP-8 (IC${}_{50}$ = 1.0 μM) and MMP-9 (IC${}_{50}$ = 50 μM) [22,26]. CP 471474 (CP) is a pyran-containing sulfonamide hydroxamate that was developed by Pfizer. It is a broad spectrum MMP inhibitor (IC${}_{50}$ values are 0.7, 0.9, 13, 16 and 1170 nM for MMP-2, MMP-13, MMP-9, MMP-3 and MMP-1, respectively) [27]. This compound also inhibits cigarette smoke-induced lung inflammation and the progression of emphysema in guinea pig models [28]. The fourth compound considered in this work, phosphoramidon (PH), lacks a hydroxamate group: it possesses instead a phosphonate, which may serve a similar role as an anionic group that can coordinate the catalytic metal ion of metalloproteinases. It was initially isolated from *Streptomyces tanashiensis* cultures, and it has been reported as an inhibitor of the metalloendopeptidase thermolysin [29] and membrane-bound zinc metalloproteinases, neprilysin (NEP) and NEP2, with a $K_{i}$ of 2 nM for both enzymes [30]. The structures of the inhibitors considered in this work are shown in Figure 2.

## 2. Results and Discussion

### 2.1. Inhibition of Proteolytic Activity

Snake venom metalloproteinases are the most abundant active component of viperid venoms [7]. These enzymes play a fundamental role in the pathogenesis of local tissue damage characteristic of viperid envenomations [31]. Specifically the metalloproteinase Batx-I from *B. atrox* venom exhibits weak hemorrhagic activity and lacks coagulant and defibrinating activity, but it is able to induce a mild myonecrosis. This metalloproteinase may contribute to the hemorrhage and local tissue damage observed in patients envenomed by *B. atrox* from Colombia [32,33], since it constitutes about the 45% of venom proteins. Batx-I hemorrhagic activity is similar to the activity reported for other P-I type SVMPs [34], but with less potency compared to P-III type SVMPs.

The delay between envenomation and antivenom injection means that antivenoms are often unable to prevent serious local tissue damage [16]. Thus, the administration of synthetic metalloproteinase inhibitors that could be administered in the field directly at the site of venom injection may represent a valid alternative to confront this difficult problem.

In this study, all the tested compounds inhibited *B. atrox* venom and Batx-I proteolytic activity in a dose-dependent fashion. Initially, all the peptidomimetics were tested at concentrations 250, 125 and 62.5 μM. The most active compounds were ARP and CP, which inhibited more than 70% enzyme activity at a concentration 62.5 μM, as shown in Figure S1 of the Supplementary Information (SI). The IC${}_{50}$ dose-response curve for the inhibition of Batx-I proteolytic activity is shown in Figure S2 of the SI. The obtained IC${}_{50}$ values for the inhibition of *B. atrox* venom and Batx-I proteolytic activity are shown in Table 1 and Table 2, respectively. The peptidomimetic CP, which has a hydroxamate zinc-binding group and a sulfonamide group was the most active for the inhibition of the proteolytic activity, followed by ARP, 4AM and PH. Together with the computational study described below, these results suggest that peptidomimetic compounds with a hydroxamate group (CP, ARP, 4AM) have a greater affinity for the metalloproteinase Batx-I, compared to compounds with a phosphonate group (PH).

### 2.2. Inhibition of Hemorrhagic Activity

Hemorrhagic activity of Batx-I was diminished by 4AM, ARP, CP and PH with IC${}_{50}$ values of 92.9, 168.3, 2.5 and 321.1 μM, respectively, for pre-incubation assays Table 2. These values are consistent with the IC${}_{50}$ values obtained for inhibition of proteolytic activity without pre-incubation. Notably, CP was the most active compound for both assays.

**Figure 3:**
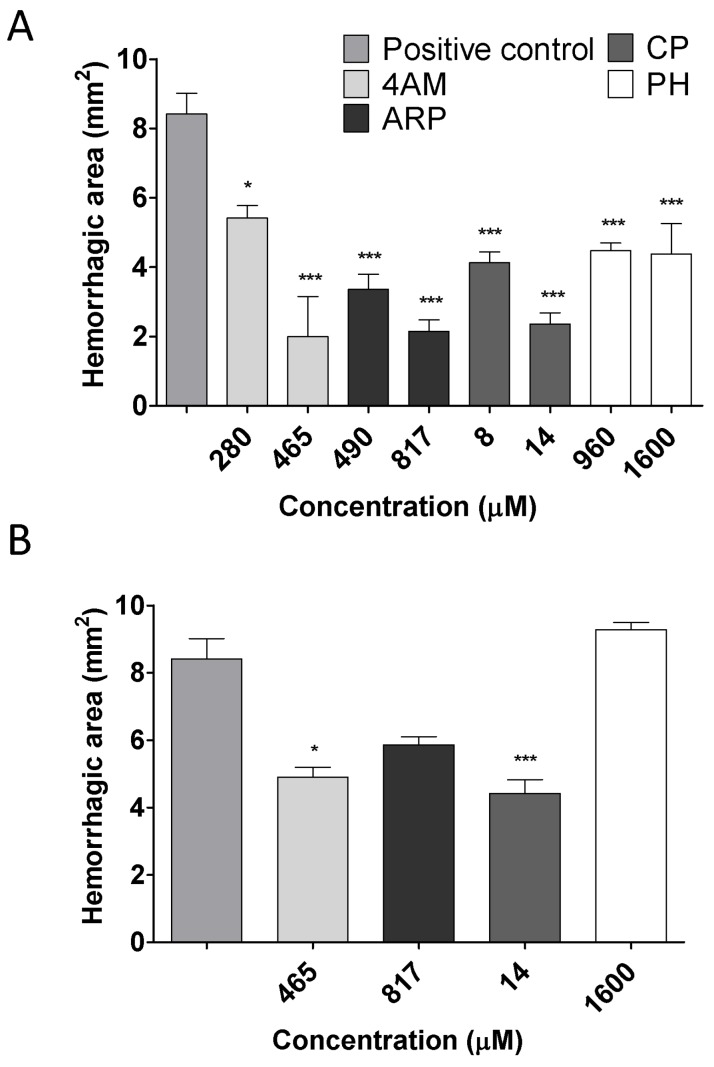
Inhibition of hemorrhagic activity induced by Batx-I with independent injection of peptidomimetics. Toxin (30 μg) was injected intradermally into the abdominal ventral region of mice and then, at two time intervals (immediately and 5 min), mice were injected at the same site of toxin injection with two different peptidomimetics concentrations, equivalent to 3 and 5 times the IC${}_{50}$ value obtained for inhibition of hemorrhagic activity with pre-incubation. Control mice were injected with toxin alone (positive control) or buffer/peptidomimetics (negative control). Two hours after toxin injection, the mice were sacrificed and their skin was removed to measure the hemorrhagic area. The results are shown as hemorrhagic area (mm${}^{2}$) (mean ± SEM). (**A**) Injection interval 0 min. (**B**) Injection interval 5 min. *** Represents statistical differences with respect to positive control with *p* value < 0.001. ** p< 0.01. * p< 0.05.

For studies with independent injection, all the studied compounds exhibited statistically significant inhibition of hemorrhagic lesions when they were administered immediately after metalloproteinase injection, in concentrations equivalent to 3 and 5 times the IC${}_{50}$ value obtained for inhibition of hemorrhagic activity with pre-incubation (Figure 3A)). When the time lapse between metalloproteinase injection and peptidomimetics administration was increased to 5 min, inhibition was significant for 4AM (*p* value < 0.05) and CP (*p* value < 0.01), using concentrations equivalent to 5 times the IC${}_{50}$ value (Figure 3B). Thus, the peptidomimetic CP appeared to be the most effective compound for neutralizing hemorrhagic activity of Batx-I when administered after metalloproteinase injection.

Previous studies with the peptidomimetic batimastat and the metalloproteinase BaP1 demonstrated significant inhibition of hemorrhage activity in independent injection assays only when this compound was administered at a concentration of 200 μM at intervals of 0 or 1 min after toxin injection [19]. Although the results obtained with CP suggest a greater inhibitory potential of this compound with respect to batimastat, the methodologies to estimate hemorrhage are different and the results should not be directly compared.

In all cases, the concentrations we used for the in vivo assays were higher than those used in studies to test the potential of these MMP inhibitors to treat cancer. For instance, ARP has been tested at concentrations of 13 nM and 25 μM [35,36] and CP471474 at 100 nM [37,38]. We did not perform cytotoxicity assays for the evaluated compounds, but all the experiments had negative controls with the inhibitors at the highest tested concentrations. No evidence of toxicity was observed in the negative controls, suggesting some level of safety for the inhibitor concentrations used. However, acute and chronic toxicity assays would need to be performed to verify the safety of these inhibitors at therapeutic concentrations.

### 2.3. Inhibition of Edema-Forming Activity

**Figure 4:**
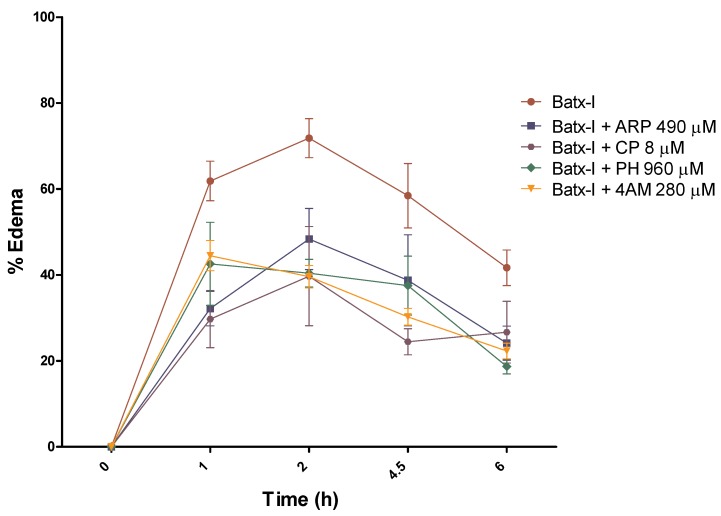
Inhibition of edema-forming activity of Batx-I by peptidomimetic compounds. 30 μg of Batx-I was pre-incubated for 30 min at 37 ${}^{\circ }$C with concentrations of compounds equivalent to three times the IC${}_{50}$ value for the inhibition of hemorrhagic activity with preincubation. Then aliquots of 50 μL were injected subcutaneously into the right footpads of mice. Controls included mice injected with the compounds alone. Edema was assessed at various time intervals by measuring the increase in footpad thickness with a caliper. Results are presented as means ± SEM (n=4). The compounds alone did not induce edema (data not shown).

Assays for inhibition of Batx-I edema-forming activity were performed by measuring the plantar pad thickness of mice injected with 30 μg of toxin and the inhibitors at various time intervals. The concentration evaluated was equivalent to three times the IC${}_{50}$ value for inhibition of hemorrhagic activity with pre-incubation. Peptidomimetic compounds demonstrated activity against Batx-I edema-forming activity. All the evaluated compounds reduced significantly Batx-I edema-forming activity at all the evaluated time intervals Figure 4. The most active compound was CP, which inhibited edema formation with *p* value < 0.01 at times of 1, 2 and 4.5 h.

These compounds’ inhibition of Batx-I edema-forming activity is consistent with their inhibition hemorrhagic activity, as well as their previously reported anti-inflammatory activity for different pathologies [39,40].

In addition, it has been described that different endogeneous MMPs, especially the latent forms of MMP-9 and MMP-2, are expressed in muscle injected with the metalloproteinase BaP1 [41]. Hence, SVMPs may activate these endogenous MMPs, which in turn may modulate various aspects of inflammation [42,43]. However, it is necessary to perform further studies to determine the effect of Batx-I on the release and activation of endogenous inflammation mediators such as MMPs and interleukin-1β and interleukin-6 and the inhibitory potential of studied peptidomimetics.

### 2.4. Computational Analysis of Inhibitor–BaP1 Interactions

**Figure 5:**
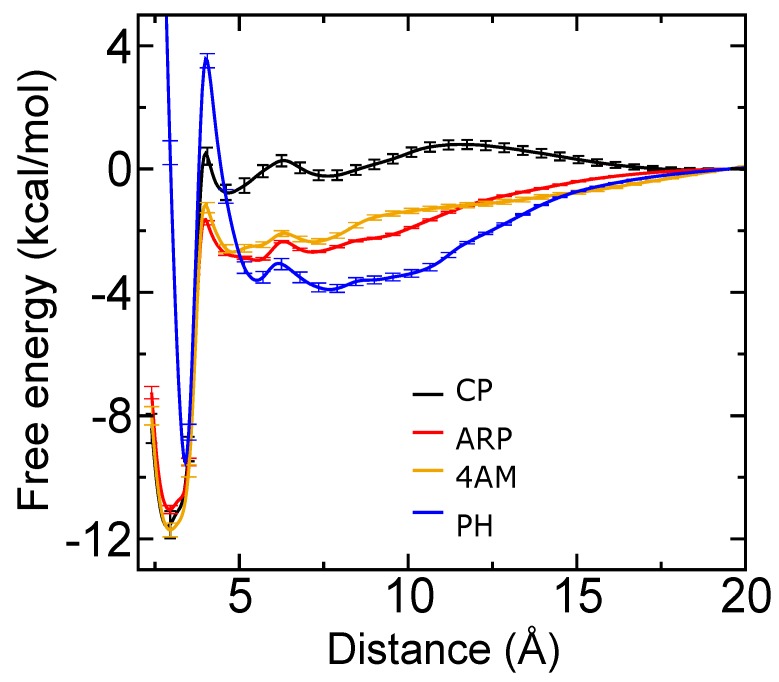
Potential of mean force as a function of distance between the hydroxamate (4AM, ARP, CP) or phosphonate group (PH) and the Zn${}^{2+}$ ion obtained from adaptive biasing force calculations.

To better understand the role of the hydroxamate, phosphonate, sulfonamide, and hydrophobic groups of the inhibitors in SVMP binding and zinc coordination, we applied computational techniques using an available x-ray crystal structure of BaP1 [10]. Molecular docking with AutoDock Vina did not show the expected contacts between the zinc binding groups (hydroxamate or phosphonate) and the Zn${}^{2+}$ cofactor. Therefore, we turned to explicit-solvent molecular dynamics simulation using the CHARMM force field [44,45] to study the interactions between the compounds and the metalloproteinases in a more realistic manner. An enhanced sampling technique [46] was employed to obtain the free energy (potential of mean force) as a function of the distance between the hydroxamate or phosphonate group of each compound and the Zn${}^{2+}$ ion. The resulting potential of mean force functions are shown in Figure 5.

**Figure 6:**
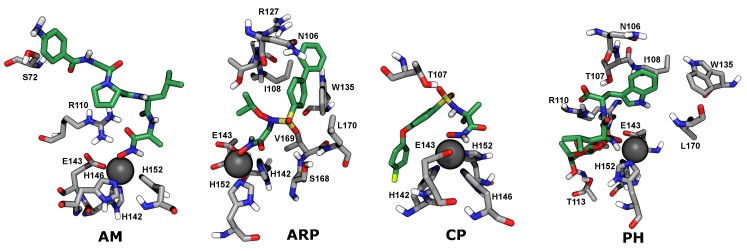
Conformations of peptidomimetic compounds bound to BaP1 associated with the free-energy minima in molecular dynamics simulations.

The potential of mean force for each compound exhibits a single free-energy minimum. In all cases, the simulations showed that binding of these compounds to BaP1 is mainly mediated by the strong electrostatic interaction between hydroxamate or phosphonate groups and the catalytic Zn${}^{2+}$ cofactor (Figure 6). Also, hydrophobic contacts between the bulky aromatic groups of each compound and amino acids and amino acid side chains located in substrate binding subsites S1 and S1${}^{\prime }$ appeared to reinforce the binding.

The hydroxamate group appears to make a major contribution to the affinity of the studied peptidomimetics for the metalloproteinase BaP1. This zinc binding group is a bidentate anion that binds the Zn${}^{2+}$ cofactor creating a distorted trigonal bipyramidal geometry around it. This geometry was previously described for a peptidomimetic inhibitor possessing a zinc-binding hydroxamate group, co-crystallized with the metalloproteinase BaP1 from the *B. asper* venom (PDB code 2W15) [10]. In addition, the effectiveness of this group is mediated by the possibility of hydrogen bond formation between the heteroatoms of the hydroxamate (specifically the NH group and the deprotonated OH group) with the side chain of Glu143 or the carbonyl of Ala111, amino acids highly conserved in all metalloproteinases of the metzincin family [47].

In the design of sulfonamide hydroxamates, such as CP and ARP, the sulfonamide group was incorporated to form hydrogen bonds and improve the enzyme–inhibitor binding by directing the hydrophobic substituent into the S1${}^{\prime }$ pocket [48]. Consistent with this, we observe hydrogen bonding between the phosphonate oxygen atoms of CP and the hydroxy group Thr107, as exemplified in Figure 6, CP. Furthermore, we also find contacts between sulfonamide-attached hydrophobic groups and side chains of residues making up the S1${}^{\prime }$ subsite, such as Ser168, Leu170, and Trp135. An example of these contacts is seen in Figure 6, ARP.

## 3. Conclusions

We found that the peptidomimetics considered here inhibited proteolytic and hemorrhagic activities of a P-I type SVMP in a dose-dependent manner. The hemorrhagic activity was reduced when the inhibitors were independently injected 5 min after the injection of the metalloproteinase. Peptidomimetics bearing a hydroxamate group (4AM, ARP, CP) were found to be more active than the compound PH, which carried a phosphonate instead. Molecular dynamics simulations supported the supposition that the hydroxamate groups of 4AM, ARP, and CP coordinated the Zn${}^{2+}$ cofactor by strong electrostatic interactions. The phosphonate of PH also coordinated the Zn${}^{2+}$ cofactor, but more weakly, which was partially responsible for the weaker predicted BaP1-binding affinity of PH as compared to the 4AM, ARP, and CP.

## 4. Materials and Methods

### 4.1. Venom

A venom pool was obtained by manual extraction from ten *B. atrox* adult specimens from the department of Meta (southwest region of Colombia) kept in captivity at the University of Antioquia serpentarium (Medellin, Colombia). The venom was centrifuged at 800× *g* for 15 min and the supernatant was lyophilized and stored at −70 ${}^{\circ }$C until use.

### 4.2. Chemicals and Reagents

The compounds 4-aminobenzoyl-Gly-Pro-D-Leu-D-Ala hydroxamic acid, ARP100, CP471474 and phosphoramidon, were purchased from Santa Cruz Biotechnology, Dallas, TX, USA. They were diluted in 1% DMSO in buffer. Other reagents used in this work were of the highest purity available from MilliporeSigma, St. Louis, MO, USA.

### 4.3. Animals

Swiss Webster mice, 18–20 g body weight, were used for the in vivo assays. All experiments were conducted in accordance with guidelines of the Universidad de Antioquia Ethics Committee (Acta No. 90, August 2014).

### 4.4. Purification of Metalloproteinase Batx-I

The metalloproteinase Batx-I was isolated from *B. atrox* venom. The toxin was purified following established methods [32]. The toxin purity was judged by RP-HPLC and SDS-PAGE [49]. Batx-I was lyophilized and stored at −20 ${}^{\circ }$C until its use.

### 4.5. Inhibition of Proteolytic Activity

Proteolytic activity was assayed on fluorescein conjugates of gelatin using the EnzCheck Gelatinase/Collagenase assay kit (Molecular Probes Inc., Eugene, OR, USA) following the protocol described by [33]. Aliquots of 80 μL of the compounds at different concentrations dissolved in buffer (0.05 M Tris-HCl, 0.15 M NaCl, 5 mM CaCl${}_{2}$, 0.2 mM sodium azide) were added to each well of a 96-well plate. Then, 20 μL of substrate (6.25 μg/μL) followed by 100 μL of the active enzyme (1 μg/μL) or venom (2 μg/μL) was added and the fluorescence intensity was measured by a Synergy HT Multi-Mode Microplate Reader (BioTekInstruments, Inc.; Winooski, VT, USA) for excitation at 485 nm and emission detection at 515 nm at each minute for 60 min. Initially, inhibitor concentrations were 250, 125, and 62.5 μM were tested. Subsequently, to better delineate the dose–response curve, we tested a few further concentration values specific to each inhibitor (see Figure S2 of the SI) Each reaction was performed in quadruplicate. The decrease in fluorescence compared with the enzyme activity (positive control) was recorded to determine percent inhibition.

### 4.6. Inhibition of Hemorrhagic Activity

Hemorrhagic activity was quantitatively determined following the method adapted by [50]. Groups of four mice were injected intradermally with different concentrations of each inhibitor and the metalloproteinase (30 μg) previously pre-incubated at 37 ${}^{\circ }$C for 30 min. The metalloproteinase alone was used as positive control and each compound alone at the highest evaluated concentration were used as negative controls. After 2 h, the animals were euthanized by CO${}_{2}$ inhalation, the skin was removed and diameters of the hemorrhagic lesions were measured. The evaluated concentrations were in the interval 25–400 μM for 4AM and ARP, 0.625–20 μM for CP, and 25–500 μM for PH, following a series of two-fold dilutions in these ranges.

For independent injection experiments, groups of four mice were injected intradermally with 30 μg of Batx-I and, subsequently, the compounds were injected at time intervals of 0 and 5 min in concentrations equivalent to three and five times the IC${}_{50}$ value obtained for experiments with pre-incubation.

### 4.7. Inhibition of Edema-Forming Activity

Edema was quantified in the footpad of mice by measuring the increase of thickness with a caliper. The metallopoteinase Batx-I (30 μg) was pre-incubated for 30 min at 37 ${}^{\circ }$C with a concentration equivalent to 3 times the ${\mathrm{IC}}_{50}$ value for the inhibition of hemorrhagic activity with preincubation. Later, 50 μL of the toxin-inhibitor mixture was injected subcutaneously into the right footpads of mice. The same controls described above were included. Edema was assessed after 1, 2, 4 and 6 h by measuring the footpad thickness with a caliper and expressed as percent of edema relative to the diameter of left footpad injected with saline solution. Each experiment was performed by quadruplicate.

### 4.8. Molecular Models

The software Avogadro 1.90.0 was used to build the compounds structure and to optimize its conformation by an energy minimization process based on the MMF94 force field. The structure of the metalloproteinase BaP1 from *B. asper* venom from Costa Rica (PDB code 2W15) [10] was used for computational studies since the 3D structure of Batx-I is not available. However, these SVMPs have comparable biological activities and portions of their sequences are identical [32]. The software AutoDock Vina (Scripps Research Institute, San Diego, CA, USA) was used to dock the studied peptidomimetics into BaP1 active site.

### 4.9. Molecular Dynamics

The compounds were parameterized with the CHARMM General Force Field using the ParamChem web interface [44,51]. The structure of each compound was built according to its protonation state at physiological pH. Thus, the oxygen atom of hydroxyl group of hydroxamate group was deprotonated (ionized). The metalloproteinase was represented in the simulations by the CHARMM36m force field for proteins [45,52] and constructed with the CHARMM-GUI server [53,54]. Conventional molecular dynamics simulations were performed with NAMD 2.13 [55] and analyzed with VMD 1.9.4a37 [56]. Lennard-Jones interactions were calculated with a 12 Å cutoff. The pressure was maintained at 101.325 kPa using the Langevin piston method. The temperature was maintained at 310.15 K using a Langevin thermostat with a damping parameter of 1 ps${}^{-1}$. All simulations were performed with mass repartitioning of ligand and protein hydrogen atoms [57]. The mass distribution of water molecules was not altered. Electrostatic interactions were calculated using the particle-mesh Ewald method [58] with a grid spacing <1.2. Water molecules were represented by the TIP3P model [59]. Sodium and chloride ions (0.15 M NaCl) were added to the aqueous phase. Additional ions were added to obtain charge neutrality.

### 4.10. Free Energy Calculations

The potentials of mean force were calculated by a hybrid of metadynamics and extended adaptive biasing force (meta-eABF) [46] using the implementation provided in the Colvars module of NAMD [60]. This algorithm uses the extended ABF method [61,62,63] to reduce free-energy barriers and calculate free energies (by integrating the potential of mean force), while a history-dependent potential term (metadynamics) [64] drives evolution of the system into less well-sampled regions of the transition coordinate.

To calculate the binding free energy of the peptidomimetics to the metalloproteinase BaP1 active site, the radial transition coordinate *r* was defined as $r=\sqrt{{\left(X-X_{0}\right)}^{2}+{\left(Y-Y_{0}\right)}^{2}+{\left(Z-Z_{0}\right)}^{2}}$ where (X,Y,Z) is the center of mass of the hydroxamate group for AM, ARP and CP, or phosphoramidate group for PH and $\left(X_{0},Y_{0},Z_{0}\right)$ is the center of mass of the Zn${}^{+2}$ ion. Meta-eABF was applied along the transition coordinate on the interval 1.8 ≤r≤ 20.0 Å using a bin size of 0.05 Å. A cylindrical restraint was applied to define the unbinding pathway, similar to the restraint used in funnel metadynamics [65]. The standard binding free energy was calculated by:

(1) $$\Delta G^{\circ }=-kT\ln\left(\pi R_{0}^{2}C_{0}\int dr\;\exp\left[-\beta \Delta G\left(r\right)\right]\right)$$

where $\beta =\frac{1}{k_{B}T}$ is the inverse thermal energy, $R_{0}$ is the radius of the restraint cylinder and $C_{0}$ is the standard concentration (1/(1660.5389 Å${}^{3}))$. Four ABF independent calculations were performed using the simulation conditions previously described. Statistical uncertainties in free energies were inferred from the differences between the four independent calculations [33].

## Figures and Tables

**Table 1:** Inhibition of *B. atrox* venom proteolytic activity. ${}^{a}$ IC${}_{50}$ values for the inhibition of *B. atrox* venom proteolytic activity on gelatin in assays (see Figure S1 of the SI) without pre-incubation. CI corresponds to the confidence interval.

Compound	IC${}_{50}$ (μM) ${}^{a}$	CI (95%) (μM)
4AM	408.3	328.8–507.1
ARP	55.4	43.1–71.1
CP	8.9	7.6–10.5
PH	793.2	450.6–1396.0

**Table 2:** Inhibition of Batx-I proteolytic and hemorrhagic activities. ${}^{a}$ IC${}_{50}$ for the inhibition of Batx-I proteolytic activity on gelatin without pre-incubation. ${}^{b}$ IC${}_{50}$ for the inhibition of Batx-I hemorrhagic activity with pre-incubation.

Compound	IC${}_{50}$ (μM) ${}^{a}$	CI (95%) (μM)	IC${}_{50}$ (μM) ${}^{b}$	CI (95%) (μM)
4AM	87.1	72.6–104.4	92.9	79.3–108.8
ARP	36.5	29.5–45.2	163.8	156.1–171.8
CP	11.6	9.3–11.3	2.5	3.1–3.0
PH	358.3	327.0–392.0	321.1	212.7–484.9

## Supplementary Materials

The following are available online at https://www.mdpi.com/2072-6651/12/1/8/s1, Figure S1: Fluorescence intensity in the experiment to measure the proteolytic activity of Batx-I in the presence of the inhibitors, Figure S2: Inhibition of Batx-I proteolytic activity as a function of concentration.

## Abbreviations

The following abbreviations are used in this manuscript:

ABF	Adaptive biasing force
Colvars	Collective variables
eABF	Extended adaptive biasing force
meta-eABF	Extended adaptive biasing force with sampling enhanced by metadynamics
MMPs	Matrix metalloproteinases
SVMPs	Snake venom metalloproteinases
