# Supplementary material for: Potential of Matrix Metalloproteinase Inhibitors for the Treatment of Local Tissue Damage Induced by a Type P-I Snake Venom Metalloproteinase
Source: Toxins (Basel). 2019 Dec 20;12(1):8. doi: 10.3390/toxins12010008 (PMC7020480; doi:10.3390/toxins12010008)
Supplement: Supplementary file 1 [file toxins-12-00008-s001.pdf]

Supplementary Materials: Potential of Matrix Metalloproteinase Inhibitors for the Treatment of Local Tissue Damage Induced by a Type P-I Snake Venom Metalloproteinase

Lina María Preciado, Jaime Andrés Pereañez and Jeffrey Comer

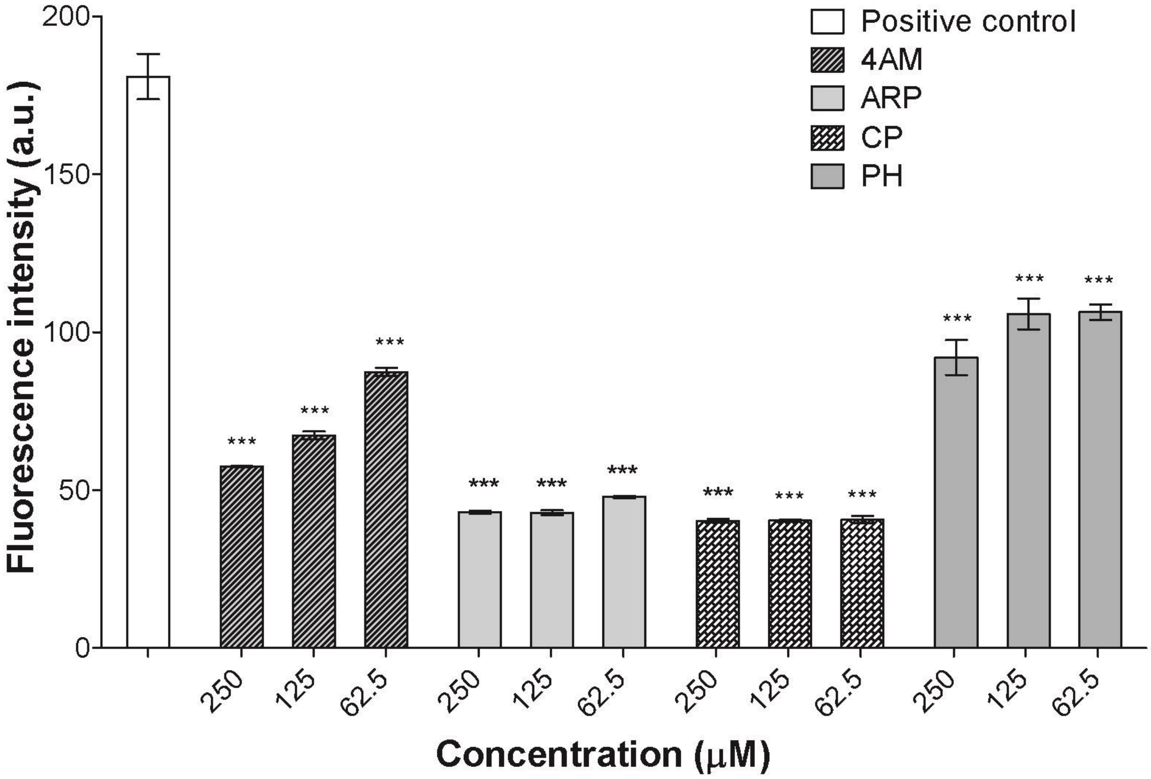

Figure S1. Fluorescence intensity in the experiment to measure the proteolytic activity of Batx-I in the presence of the inhibitors.

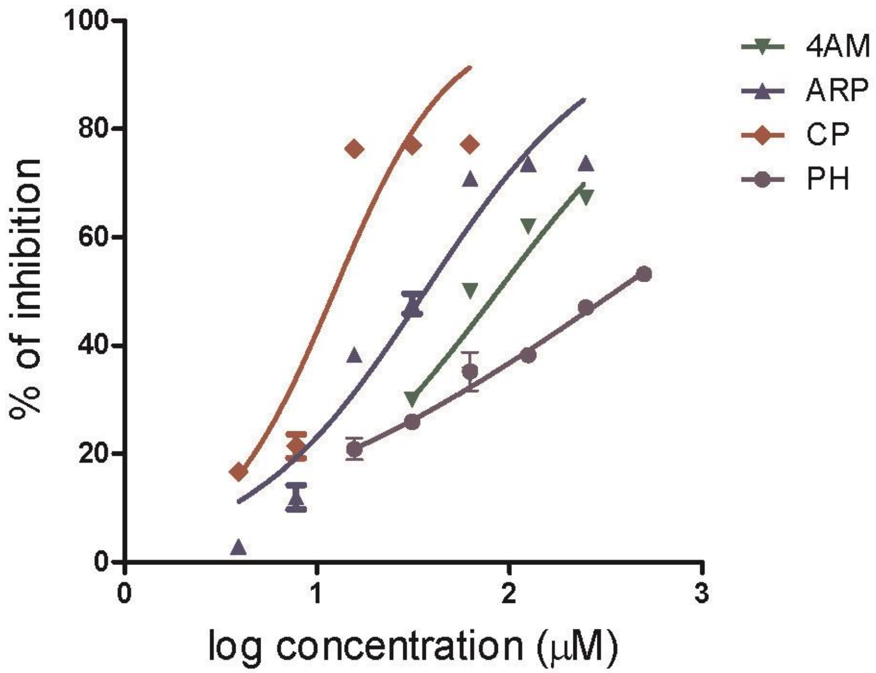

Figure S2. Inhibition of Batx-I proteolytic activity as a function of inhibitor concentration.
